# Influence of barley inclusion method and protease supplementation on growth performance, nutrient utilisation, and gastrointestinal tract development in broiler starters

**DOI:** 10.1016/j.aninu.2021.06.008

**Published:** 2021-09-23

**Authors:** Lindon M. Tari, Nipuna Perera, Faegheh Zaefarian, M. Reza Abdollahi, Aaron J. Cowieson, Velmurugu Ravindran

**Affiliations:** a Monogastric Research Centre, School of Agriculture and Environment, Massey University, Private Bag 11 222, Palmerston North, 4442, New Zealand; b Department of Animal Science, Faculty of Agriculture, University of Peradeniya, Peradeniya, 20400, Sri Lanka; c DSM Nutritional Products, Wurmisweg 576, Kaiseraugst, Switzerland

**Keywords:** Barley, Broiler, Particle size, Protease, Whole grain

## Abstract

The influence of the method of barley inclusion (fine, coarse and whole barley) in a wheat-based diet and protease supplementation (0 and 0.20 g/kg) on growth performance, nutrient utilisation and gastrointestinal tract development of broilers (d 1 to 21) was evaluated in a 3 × 2 factorial arrangement. Whole barley (WB) grains were ground in a hammer mill to pass through the screen sizes of 2.5 and 8.0 mm to achieve fine (FB) and coarse (CB) barley particle sizes, respectively. A total of 288, one-day-old male broilers were allotted to 36 cages (6 cages/treatment; 8 birds/cage). There was no significant (*P* > 0.05) interaction between barley inclusion method and protease for any growth performance or nutrient utilisation parameters. Birds fed diets containing CB and WB showed higher (*P* < 0.05) weight gain, and digestibility of dry matter, nitrogen, calcium, gross energy, and ileal digestible energy compared to those fed FB diets. Compared to the birds fed FB diets, feed per gain was lower (*P* < 0.05) in birds fed diets made of WB. Fat digestibility of the birds fed CB was higher (*P* < 0.05) than those fed FB and WB birds. Compared to FB and CB diets, inclusion of WB resulted in heavier (*P* < 0.05) gizzards but reduced (*P* < 0.05) gizzard pH. Supplemental protease, however, had no effects (*P* > 0.05) on growth performance and nutrient utilisation, most likely due to the well balanced digestible amino acids and high inherent digestibility of protein in the basal diet, and/or the presence of exogenous carbohydrase and phytase. In conclusion, the present results showed that the inclusion of coarsely ground and whole barley in a wheat-based diet can enhance nutrient and energy utilisation and is beneficial to the growth performance of young broilers.

## 1 Introduction

The prediction that conventional cereals such as maize and wheat will not meet the future demands of animal feed industry, especially with the fast-growing poultry production poses immense pressure on the feed market and encourages the evaluation of alternative feed ingredients that have promise for use in poultry diets (Abdollahi and Ravindran, 2019). There is also increasing demand for locally grown cereals and grain legumes to reduce the dependance on imports. Locally grown ingredients will also encourage biodiversity and reduce the carbon footprint of poultry production. Barley (*Hordeum vulgare* L.) is one such ingredient. The use of barley, however, remains low in poultry diets due to the presence of anti-nutritive, soluble non-starch polysaccharides (NSP; Jacob and Pescatore, 2012). The use of NSP-degrading enzymes in barley-based diets has become a norm to overcome the adverse effects of NSP on nutrient utilisation and bird performance, and the potential for improving the efficacy of supplemental enzymes through optimising the physical texture of diets has been increasingly recognised (Amerah et al., 2011a; Amerah, 2015). Feeding highly processed pelleted diets have been shown to suppress the foregut functionality of broiler chickens (Abdollahi et al., 2013a, 2019; Rodrigues and Choct, 2018). This concern has raised the interest on methods to restore the structure of the pelleted diets by the inclusion of insoluble fibre (Hetland et al., 2004), coarse cereal particles (Amerah et al., 2007a; Abdollahi et al., 2019) or whole grains (Singh et al., 2014a) in broiler diets to improve the physical microstructure. Manipulation of grain particle size and whole grain inclusion are comparatively feasible, even in commercial poultry production, and thus remain an attractive strategy than insoluble fibre inclusion which might dilute the diet.

Pelleting can enhance the feeding value of alternative feed ingredients, such as barley, in poultry diets mostly through improved palatability and break-down of cell wall matrix resulting in a greater accessibility of encapsulated nutrients to digestive enzymes (Abdollahi et al., 2013a). While the optimum inclusion level of barley in pelleted broiler diets has been evaluated (Perera et al., 2019b), the most favourable method of barley inclusion in broiler diets remains unexplored. Perera et al. (2019b) suggested that the optimum inclusion level of a normal-starch hulled (NSH) barley in wheat-based diets to be 283 g/kg of the diet. This inclusion level was used in the current study to formulate the experimental diet. Previous studies have compared different particle sizes of barley (Perera et al., 2020), flaked (Svihus et al., 1997) and whole barley (Moss et al., 2017). However, to the authors' knowledge, this study is the first one evaluating different barley particle sizes and whole barley inclusion in a single experiment.

With the growing scrutiny on animal welfare and environmental sustainability, exogenous protease has attracted attention for its role in reducing nitrogen (N) emissions from commercial poultry production. Moreover, it has been demonstrated that supplemental protease can reduce diet cost by enhancing dietary protein utilisation (Cowieson and Roos, 2016). In barley-based diets, exogenous protease has been evaluated in multi-component enzyme mixtures (Villamide et al., 1997; Józefiak et al., 2010). However, these multi-component enzyme mixtures were commonly described as carbohydrases and, protease activity *per se* has not been explicitly examined (O'Neill et al., 2014). The recent introduction of mono-component protease has allowed an enzyme-specific interpretation of results encouraging the evaluation of mono-component protease for protein sources as well as cereals. While the protease effect has been evaluated in maize- (Kocher et al., 2003; Barekatain et al., 2013; Liu et al., 2015), sorghum- (Selle et al., 2013; Liu et al., 2015) and wheat- (Kalmendal and Tauson, 2012; Liu et al., 2015) based diets, such studies with barley are limited.

In addition to enhancing the amino acid (AA) digestibility, the extra-proteinous effects of protease particularly on starch, fat and energy utilisation have been reported (Kalmendal and Tauson, 2012; Selle et al., 2013). Nutrients in feed ingredients are present in a complex matrix comprising of starch, protein, lipid, NSP, minerals and vitamins. The extra-proteinous effect of protease is mainly attributed to the release of nutrients due to the changes in the macrostructure of the nutrient matrix following proteolysis (Cowieson and Roos, 2016). The extent of grinding can also expose the interior of endosperm cells in cereals to enzymatic attack and, therefore, it is plausible to assume that barley particle size might interact with protease on the extent that protease can breakdown the feed matrix. Despite the potential for this interactive effect, no study has examined the effects on nutrient digestibility and growth performance. It is therefore hypothesised that the method of barley inclusion can influence the efficacy of supplemental protease on growth performance and nutrient utilisation of broiler starters fed wheat-based diet. Accordingly, the present study was initiated to investigate the possible interaction between barley inclusion method (BIM; fine, coarse and whole barley) and protease supplementation in a wheat-based diet on performance and, nutrient and energy utilisation in broiler chickens.

## 2 Materials and methods

### 2.1 Enzymes

A multi-component NSP-degrading enzyme, Ronozyme Multigrain (produced by *Trichoderma reesei,* also known as *Trichoderma longiabrachiatum*), a mono-component bacterial protease (Ronozyme ProAct (GT), 15,000 U/kg feed) and Ronozyme HiPhos were obtained from DSM Nutritional Products, East Wagga Wagga, Australia. The activities of endo-1,4-β-glucanase, endo-1,3 (4)-β-glucanase and endo-1,4-β-xylanase in Ronozyme Multigrain were 800 g, 700 and 2,700 U/g, respectively. One unit of xylanase activityis defined as the quantity of enzyme that releases 1.0 μmol of reducing moieties from 1.5% arabinoxylan per minute at pH 5.0 and incubation temperature of 40 °C for 20 min. One unit of β-glucanase activity is defined as the quantity of enzyme that releases 1.0 μmol of reducing moieties from 1.5% β-glucan per minute at pH 5.0 at incubation temperature of 40 °C for 20 min. Ronozyme HiPhos is a granular 6-phytase preparation expressed by submerged fermentation of *Aspergillus oryzae* and contains >10,000 phytase U/g. One unit of phytase is defined as the quantity of enzyme which liberates 1.0 μmol of inorganic phosphate per minute from 5.0 μmol/L sodium phytate at pH 5.5 at 37 °C. One protease unit is defined as the amount of enzyme that releases 1.0 mmol of p-nitroaniline from 1.0 mmol/L substrate (Suc-Ala-Ala-Pro-Phe-pNA) per minute at pH 9.0 and 37 °C. The activities of protease, phytase, endo-1,3(4)-β-glucanase and endo-1,4-β-xylanase in diet samples were measured at Biopract GmbH, Berlin, Germany. The enzyme recovery was calculated as the percentage of measured enzyme activity in the diet to the expected enzyme activity estimated from the amount and minimum activity (DSM Nutritional Products Ltd, 2013) of enzymes added to the diets.

### 2.2 Diets

Normal-starch hulled (NSH) barley (cultivar, Fortitude), obtained from a seed multiplication company (Luisetti Seeds Ltd., Rangiora, New Zealand), was ground in a hammer mill to pass through screen sizes of 2.5 and 8.0 mm to achieve fine and coarse barley particle sizes, respectively. Wheat was obtained from a local commercial supplier and ground to a size of 3.0 mm. The basal diet was formulated to meet the Ross 308 strain recommendations for major nutrients (Ross 2019, Table 1). The nutrient composition, nitrogen-corrected apparent metabolizable energy (AMEn) and standardised digestible AA contents of barley and wheat samples, determined in a previous study (Perera et al., 2019a), were used to formulate the basal diet. Barley was included at 283 g/kg of the diet based on the recommendation by Perera et al. (2019b) as the optimum inclusion level for NSH barley type in wheat-based diets. A completely randomised design was used in this study, with a 3 × 2 factorial arrangement of 6 treatments, which included 3 different methods of barley inclusion [fine (FB), coarse (CB) and whole (WB) barley] and 2 protease (protease; Ronozyme ProAct GT) enzyme supplementation (0 and 0.20 g/kg). An NSP-degrading enzyme (Ronozyme Multigrain) was added in all diets at a rate of 0.15 g/kg of each diet. Phytase (Ronozyme HiPhos) was also used in all diets at a rate of 0.1 g/kg diet and phytase matrix values [(1.5 g/kg non-phytate phosphorus and 1.8 g/kg calcium (Ca)] were used in diet formulation. To determine the ileal nutrient digestibility, titanium dioxide (TiO_2_, Merck KGaA, Darmstadt, Germany) was added (5.0 g/kg) as an indigestible marker. Diets were mixed in a single-screw paddle mixer. Following mixing, all diets were steam-conditioned at 70 °C for 30 s and pelleted in the pellet mill (Model Orbit 15; Richard Sizer Ltd., Kingston-upon-Hull, UK) equipped with a die ring with 3 mm holes and 35 mm thickness. Representative diet samples were collected after pelleting for chemical analysis and determination of particle size distribution and pellet durability.

**Table 1:** Composition, calculated and analyzed values (g/kg, as fed) of the basal broiler starter diet (d 1 to 21).

Item	Inclusion
Ingredients, g/kg	
Wheat	314
Barley (Normal-starch hulled barley)	283
Soybean meal	297
Maize gluten meal	50.0
Soybean oil	16.4
Di-calcium phosphate	11.0
Limestone	8.70
L-Lysine HCl	3.45
DL-Methionine	2.20
L-Threonine	1.30
Sodium chloride	2.10
Sodium bicarbonate	3.60
Titanium dioxide^1^	5.00
Vitamin and mineral premix^2^	2.00
Ronozyme Multigrain^3^	0.15
Ronozyme HiPhos^4^	0.10
Calculated analysis, g/kg	
Apparent metabolizable energy, MJ/kg	11.9
Crude protein	225
Digestible methionine	5.80
Digestible methionine + cysteine	9.00
Digestible lysine	12.2
Digestible threonine	8.20
Crude fat	30.5
Crude fibre	37.8
Calcium	9.60
Non-phytate phosphorus	4.80
Sodium	2.00
Chloride	2.00
Analysed values, g/kg	
Dry matter	908
Gross energy, MJ/kg	16.8
Crude protein, N × 6.25	232
Fat	28.2
Starch	326
Calcium	9.10
Phosphorus	5.90

1 Merck KGaA, Darmstadt, Germany.

2 Supplied per kilogram of diet: antioxidant, 100 mg; biotin, 0.2 mg; calcium pantothenate, 12.8 mg; cholecalciferol, 60 μg; cyanocobalamin, 0.017 mg; folic acid, 5.2 mg; menadione, 4 mg; niacin, 35 mg; pyridoxine, 10 mg; trans-retinol, 3.33 mg; riboflavin, 12 mg; thiamine, 3.0 mg; dl-α-tocopheryl acetate, 60 mg; choline chloride, 638 mg; Co, 0.3 mg; Cu, 3.0 mg; Fe, 25 mg; I, 1 mg; Mn, 125 mg; Mo, 0.5 mg; Se, 200 μg; Zn, 60 mg.

3 Ronozyme Multigrain (800 U/g endo-1,4-β- glucanase, 700 U/g endo-1,3 (4)-β-glucanase and 2,700 U/g endo-1,4-β-xylanase. One unit of xylanase is defined as the quantity of enzyme that releases 1 μmol of reducing moieties from 1.5% arabinoxylan per minute at pH 5.0 and incubation temperature of 40 °C for 20 min. One unit of β-glucanase is defined as the quantity of enzyme that releases 1 μmol of reducing moieties from 1.5% β-glucan per minute at pH 5.0 at incubation temperature of 40 °C for 20 min.

4 Ronozyme HiPhos, DSM Nutritional Products, Kaiseraugst, Switzerland (1,000 phytase U/kg diet). One unit of phytase is defined as the activity of enzyme that releases 1.0 μmol of inorganic phosphorus per minute from 5.0 μmol/L sodium phytate at pH 5.5 at 37 °C.

### 2.3 Determination of particle size distribution, pellet durability and hardness

Dry sieving was used to determine the particle size distribution of ground barley (2.5 and 8.0 mm) using the method described by Baker and Herrman (2002). Briefly, ground barley samples (100 g; two replicates per particle size) were passed through a set of 6 (2,000, 1,000, 500, 250, 125, 63 μm) steel sieves (Endecotts Ltd., London, UK) on the shaker for 5 min. The amount of sample retained on each sieve was determined and the geometric mean diameter (GMD) and geometric standard deviation (GSD) was calculated for each sample.

Particle size distribution of the 3 basal diets (diets containing FB, CB and WB), both in mash and pellet forms, were determined by wet sieving method described by Lentle et al. (2006). Two samples of each diet (100 g; two replicates per diet) either in mash or pellet form were weighed. The first sample of each diet was dried at 80 °C in a forced draft oven for 3 d to determine the dry matter (DM) content, and the second sample was soaked in 400 mL water and was allowed to stand for 2 h prior to sieving to ensure adequate hydration. The same sieve sizes used in the dry sieving method were used. The contents of each of the sieves were subsequently washed onto dried, preweighed filter papers, dried in a forced draft oven at 80 °C for 24 h, and re-weighed. The dry weight of particles retained by each sieve was expressed as a proportion of the total DM recovered.

The pellet durability index (PDI) of the diets was determined using a Holmen Pellet Tester (New Holmen NHP100 Portable Pellet Durability Tester, TekPro Ltd., Willow Park, North Walsham, Norfolk, UK) as described by Abdollahi et al. (2013b). Pellet hardness was tested in a Stable Micro Systems Texture Analyser (TA-XT Plus, Godalming, Surrey, UK) using the method described by Abdollahi et al. (2010). Fifteen pellets of similar size were selected from each diet. Each individual pellet was then inserted between a pressure piston and a bar. The force (N) needed to break the pellets was determined by increasing the pressure applied by means of the pressure piston.

### 2.4 Birds, housing, and performance data

Experimental procedures were conducted in accordance with the guidelines of Massey University Animal Ethics Committee and complied with the Revised New Zealand Code of Practice for the Care and Use of Animals for Scientific Purposes. A total of 288, one-day-old male broilers (Ross 308) were obtained from a commercial hatchery, individually weighed and allocated to 36 cages in electrically heated battery brooders so that the average weight per cage was similar. Each of the 6 dietary treatments were then randomly assigned to 6 cages, each housing 8 birds. The birds were transferred to grower cages on d 11 and were fed the same diets until the end of the trial (d 21). The battery brooders and grower cages, with wire floor, were housed in an environmentally controlled room with 20 h of fluorescent illumination per day. The temperature was set to 31 °C on d 1 and was gradually reduced to 22 °C by 21 d of age. The diets were offered ad libitum and water was always available.

Body weights (BW) and feed intake (FI) were recorded on a cage basis at weekly intervals throughout the 21-d trial. Mortality was recorded daily. Feed per gain (F/G) values were corrected for the BW of any bird that died during the experimental period.

### 2.5 Determination of apparent metabolizable energy (AME)

The AME was determined using the classical total excreta collection method. Feed intake and total excreta output of each cage were quantitatively measured from d 17 to 20 post-hatches. Daily excreta collections from each cage were pooled, mixed in a blender and subsampled. Sub-samples were lyophilised (Model 0610, Cuddon Engineering, Blenheim, New Zealand), ground to pass through a 0.5-mm sieve, and stored in airtight plastic containers at 4 °C pending analysis. The diets and excreta samples were analysed for DM, gross energy (GE), and nitrogen (N).

### 2.6 Determination of coefficient of apparent ileal digestibility (CAID) of nutrients and jejunal digesta viscosity

On d 21, 6 broilers per cage were euthanised by intravenous injection (0.5 mL/kg live weight) of sodium pentobarbitone (Provet NZ Pty Ltd., Auckland, New Zealand), and ileal digesta were collected from the lower half of the ileum by gently flushing with distilled water as described by Ravindran et al. (2005). The ileum is defined as the portion of the small intestine extending from the Meckel's diverticulum to a point approximately 40 mm proximal to the ileo-caecal junction. The ileum was divided into 2 halves, and the digesta was collected from the lower half towards the ileo-caecal junction. The digesta from birds within a cage were pooled, frozen immediately and subsequently lyophilised. Diets and digesta samples were ground to pass through a 0.5-mm sieve and then stored in airtight containers at 4 °C until laboratory analysis. The diets and digesta samples were analysed for DM, titanium (Ti), GE, N, starch, fat, Ca and phosphorus (P).

The viscosity of jejunal digesta from two birds euthanised for ileal digesta collection was also measured as described by Perera et al. (2019b). The jejunum is defined as portion of small intestine extending from pancreatic loop to the Meckel's diverticulum. The jejunum was divided into 2 halves, and the digesta were collected from the lower half toward the Meckel's diverticulum. Digesta collected from each bird were centrifuged at 3,000 × *g* at 20 °C for 15 min. A 0.5 mL aliquot of the supernatant was used in a viscometer (Brookfield digital viscometer, Model DV2TLV, Brookfield Engineering Laboratories Inc., Stoughton, MA) fitted with CP-40 cone spindle with shear rates of 5 to 500/s to measure the viscosity.

### 2.7 Gizzard pH and digestive tract measurements

On d 21, two additional birds with BW closest to the mean weight of the cage were weighed and euthanised by intravenous injection of pentobarbitone solution. The gastrointestinal tract was eviscerated immediately, and the gizzard was excised. Gizzard pH was measured by inserting the probe of a digital pH meter (pH spear, Oakton Instruments, Vernon Hill, IL) in 3 different regions (proximal, middle and distal) of the gizzard. The average of the three readings was considered as the final pH value. The relative empty weights of the crop, proventriculus, gizzard, duodenum, jejunum, and ileum were determined in the same birds and reported as g/kg BW.

### 2.8 Chemical analyses

DM was determined using standard procedures (Method 930.15; AOAC, 2016). N was determined by combustion (Method 968.06; AOAC, 2016) using a CNS-200 carbon, N and sulphur auto-analyser (LECO Corporation, St. Joseph, MI). An adiabatic bomb calorimeter (Gallenkamp Autobomb, London, UK) standardised with benzoic acid was used for the determination of GE. Starch was measured using a Megazyme kit (method 996.11; AOAC, 2016) based on thermostable α-amylase and amyloglucosidase (McCleary et al., 1997). Fat was determined using the Soxtec extraction procedure for animal feed, forage, and cereal grains (Method, 2003.06; AOAC, 2016). Concentration of Ca and P were determined by inductively coupled plasma optical emission spectroscopy using a Thermo Jarrell Ash IRIS instrument. The samples were analyzed for Ti on a UV spectrometer following the method described by Short et al. (1996).

### 2.9 Calculations

All data were expressed on a DM basis, and the AME was calculated using the following formula:

AME_diet_ (MJ/kg) = [(FI × GE_diet_) – (Excreta output × GE_excreta_)]/FI .

Correction for zero N retention was made using a factor of 36.54 kJ per gram N retained in the body (Hill and Anderson, 1958):

N-corrected AME_diet_ (AMEn; MJ/kg) = AME_diet_ – (36.54 × N retention)/1,000 .

The CAID of nutrients were calculated from the dietary ratio of nutrients to Ti relative to the corresponding ratio in the ileal digesta:

CAID of nutrient = [(Nutrient/Ti)_diet_ - (Nutrient/Ti)_ileal_]/(Nutrient/Ti)_diet_ ,

where (Nutrient/Ti)_diet_ is the ratio of nutrient to Ti in the diet and (Nutrient/Ti)_ileal_ is the ratio of nutrient to Ti in the ileal digesta.

Ileal digestible energy (IDE) was calculated using the following formula: IDE (MJ/kg) = GE_diet_ × CAID of GE .

### 2.10 Statistical analysis

The data were analysed by two-way analysis of variance (ANOVA) to determine the main effects (BIM and protease) and their interaction using the General Linear Models procedure of SAS (version 9.4; SAS Institute., Cary, NC). Cage means served as the experimental unit for all data and the differences were considered to be significant at *P* < 0.05. Significant differences between means were separated by Least Significant Difference Test.

## 3 Results

### 3.1 Enzyme recovery

The average recovery of endo-1,3(4)-glucanase, endo-1,4-β-xylanase, phytase, and protease (in protease supplemented diets) were 88%, 155%, 201% and 78%, respectively.

### 3.2 Particle size distribution, pellet durability index and pellet hardness

**Fig. 1:**
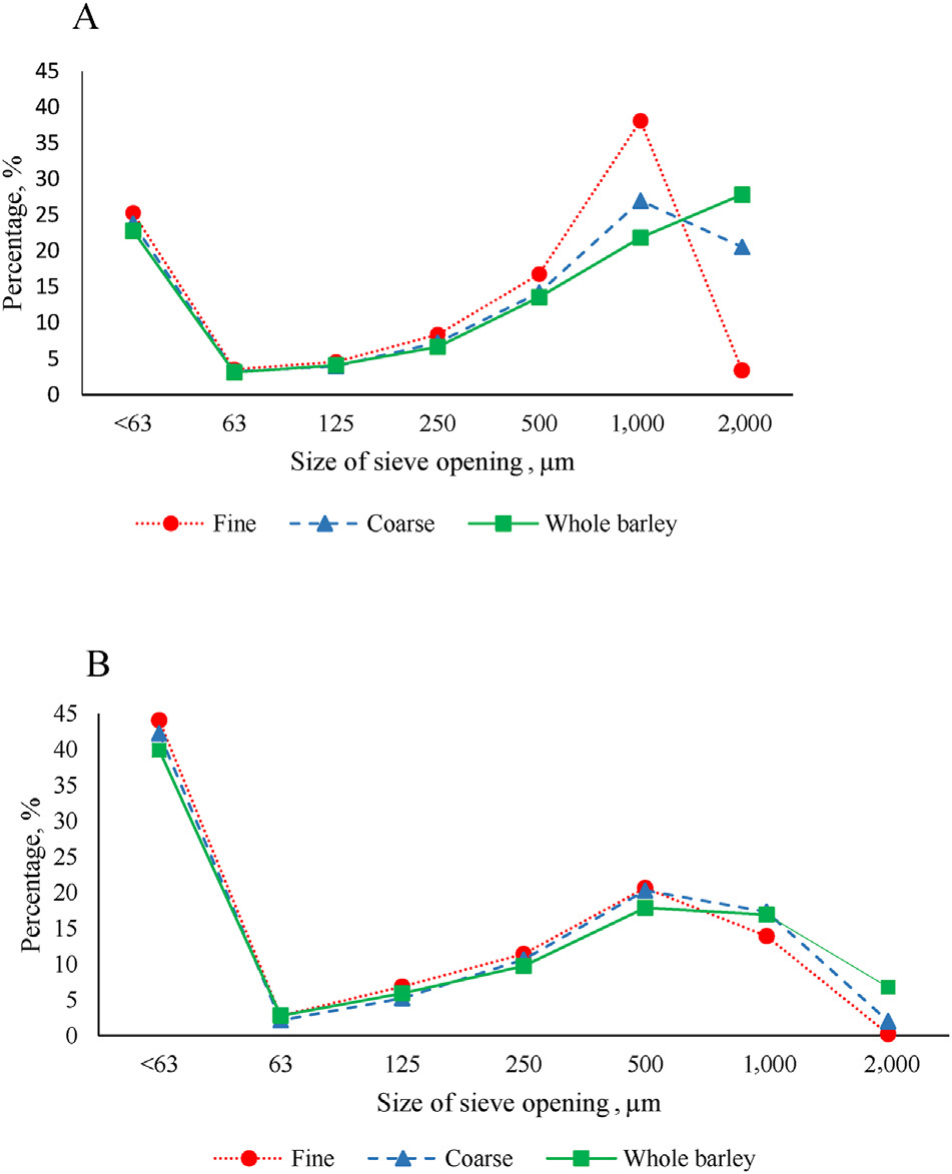
Particle size distribution of mash (A) and pelleted (B) diets.

The GMD values of barley ground through 2.5- and 8.0-mm screen sizes were determined to be 635 and 1,274 μm, respectively, with corresponding GSD values of 2.1 and 1.8 μm (Table 2). Graphic comparisons of the particle size distributions of mash and pelleted diets (Fig. 1) determined by wet sieving revealed that pelleting reduced the relative proportion of large particles (>1,000 μm) and increased that of fine particles (<63 μm) in all diets and, therefore, the differences between GMD of the three BIM were smaller in pelleted diets compared to the mash diets (Table 2). The PDI was significantly (*P* < 0.05) higher in pelleted diets made from FB than those made from CB or WB (Table 2). There was no difference (*P* > 0.05) in pellet hardness between pellets made from FB, CB or WB.

**Table 2:** Particle size distribution (percentage of retained particles on sieve) 1 and geometric mean diameter ± geometric standard deviation (GMD ± GSD) of ground barley, mash and pelleted diets, and the pellet durability index (PDI)^2^ and pellet hardness.^3^.

Item	Openings, μm								Pellet quality	
	2,000	1,000	500	250	125	63	<63	GMD ± GSD	PDI, %	Pellet hardness, N
Ground barley										
Fine	0.00	28.70	42.56	17.92	6.91	3.16	0.75	635 ± 2.1	–	–
Coarse	30.00	47.48	14.62	5.28	1.90	0.60	0.12	1274 ± 1.8	–	–
Mash diets										
Fine	3.36	38.11	16.77	8.37	4.56	3.55	25.28	399 ± 4.1	–	–
Coarse	20.57	26.99	14.21	7.24	3.99	3.21	23.79	478 ± 4.4	–	–
Whole barley	27.85	21.86	13.57	6.66	4.12	3.11	22.83	515 ± 4.5	–	–
Pelleted diets										
Fine	0.24	13.95	20.67	11.41	6.89	2.78	44.06	190 ± 3.9	79.5^a^	19.2
Coarse	2.02	17.36	20.31	10.66	5.24	2.16	42.25	217 ± 4.1	76.7^b^	20.0
Whole barley	6.78	16.92	17.90	9.74	5.94	2.82	39.90	239 ± 4.3	75.7^b^	19.5
SEM									0.57	0.79
*P-*value									0.001	0.758

^a, b^ Means in a column not sharing a common letter are significantly different (*P* < 0.05).

1 Fine and coarse grades were achieved using screen sizes of 2.5 and 8.0 mm, respectively. Each value represents the mean of 2 replicates.

2 Each value represents the mean of 6 replicates.

3 Each value represents the mean of 15 replicates.

### 3.3 Growth performance

The overall mortality rate of 2.9% was negligible and not related to treatments. No significant (*P* > 0.05) interactions were observed between the BIM and protease on broiler growth performance (Table 3). The main effect of BIM was significant (*P* < 0.01) for weight gain (WG) and F/G. Birds fed CB and WB diets had higher (*P* < 0.05) WG than those fed FB diets, by an average of 36.5 g/bird. Inclusion of WB reduced F/G compared to FB, and CB diet resulted in an intermediate value. A tendency (*P* = 0.078) was noted for BIM to affect FI. Birds fed CB and WB diets tended to consume more feed than those fed the FB diet. No significant (*P* > 0.05) differences in performance parameters were observed in response to protease supplementation.

**Table 3:** Influence of barley inclusion method^1^ and protease supplementation on weight gain, feed intake and feed per gain (F/G) of broiler starters (0 to 21 d)^2^.

Barley inclusion method	Protease	Weight gain, g/bird	Feed intake, g/bird	F/G, g feed/g gain
Fine	–	1,138	1,404	1.241
	+	1,152	1,406	1.238
				
Coarse	–	1,178	1,444	1.226
	+	1,173	1,427	1.221
				
Whole barley	–	1,183	1,433	1.212
	+	1,190	1,431	1.211
SEM		12.8	14.4	0.0083
Main effects				
Barley inclusion method				
Fine		1,145^b^	1,405	1.239^a^
Coarse		1,176^a^	1,436	1.223^ab^
Whole barley		1,187^a^	1,432	1.212^b^
				
Protease	–	1,167	1,427	1.226
	+	1,172	1,421	1.223
*P*-value				
Barley inclusion method		0.009	0.078	0.009
Protease		0.614	0.633	0.639
Barley inclusion method × Protease		0.765	0.779	0.960

^a, b^ Means in a column not sharing a common letter are significantly different (*P* < 0.05).

1 Fine and coarse grades were achieved using screen sizes of 2.5 and 8.0 mm, respectively.

2 Each value represents the mean of 6 replicates (8 birds per replicate).

### 3.4 Nutrient digestibility and energy utilisation

Neither the main effect of protease inclusion nor the BIM × protease interaction was significant (*P* > 0.05) for nutrient digestibility and energy utilisation (Table 4). The BIM had a significant effect (*P* < 0.05 to 0.01) on the CAID of DM, N, fat, Ca, P, GE and IDE. Incorporation of CB and WB increased CAID of DM, N, Ca, GE, and IDE compared to FB diets. Fat digestibility was improved only in CB diets, and WB inclusion impaired P digestibility compared to FB and CB diets. Neither the effect of BIM or protease nor the interaction was significant (*P* > 0.05) for AMEn.

**Table 4:** Influence of barley inclusion method^1^ and protease supplementation on the coefficient of apparent ileal digestibility (CAID) of dry matter (DM), nitrogen (N), starch, fat, calcium (Ca), phosphorus (P), gross energy (GE), and ileal digestible energy (IDE) and N-corrected apparent matabolizable energy (AMEn) in broiler starters.

Barley inclusion method	Protease	CAID^1^							Energy utilisation	
		DM	N	Starch	Fat	Ca	P	GE	IDE, MJ/kg DM	AMEn, MJ/kg DM
Fine	–	0.578	0.713	0.957	0.715	0.345	0.648	0.606	11.20	12.70
	+	0.597	0.726	0.954	0.749	0.353	0.664	0.623	11.52	12.49
										
Coarse	–	0.628	0.752	0.946	0.829	0.456	0.672	0.653	12.07	12.65
	+	0.633	0.762	0.955	0.841	0.451	0.656	0.660	12.20	12.61
										
Whole barley	–	0.634	0.778	0.957	0.725	0.455	0.595	0.659	12.18	12.77
	+	0.632	0.786	0.956	0.728	0.453	0.591	0.659	12.18	12.81
SEM		0.017	0.016	0.005	0.041	0.037	0.020	0.017	0.320	0.126
Main effects										
Barley inclusion method										
Fine		0.588^b^	0.720^b^	0.956	0.732b	0.349^b^	0.656^a^	0.615^b^	11.36^b^	12.60
Coarse		0.630^a^	0.757^a^	0.950	0.835a	0.453^a^	0.664^a^	0.657^a^	12.13^a^	12.63
Whole barley		0.633^a^	0.782^a^	0.957	0.726b	0.454^a^	0.593^b^	0.659^a^	12.18^a^	12.79
										
Protease	–	0.613	0.748	0.953	0.756	0.418	0.638	0.640	11.82	12.71
	+	0.620	0.758	0.955	0.772	0.419	0.637	0.647	11.96	12.64
*P-*value										
Barley inclusion method		0.023	0.002	0.340	0.020	0.011	0.002	0.026	0.025	0.293
Protease		0.608	0.446	0.660	0.628	0.986	0.926	0.579	0.585	0.490
Barley inclusion method × Protease		0.823	0.984	0.388	0.929	0.983	0.714	0.885	0.887	0.614

^a, b^ Means in a column not sharing a common letter are significantly different (*P* < 0.05).

^2^Each value represents the mean of 6 replicates (6 birds per replicate).

1 Fine and coarse grade were achieved using screen sizes of 2.5 and 8.0 mm, respectively.

### 3.5 Digestive tract measurements, gizzard pH and jejunal digesta viscosity

Neither the effect of protease supplementation nor the interaction (*P* > 0.05) between BIM and protease was observed for the relative weights of any digestive organs, gizzard pH and jejunal digesta viscosity (Table 5). Compared to FB diets, inclusion of WB reduced (*P* < 0.05) gizzard pH, and the relative weights of crop, proventriculus, jejunum and ileum, but resulted in greater (*P* < 0.05) gizzard weights than FB and CB diets. The BIM had no effect (*P* > 0.05) on the jejunal digesta viscosity.

**Table 5:** Influence of barley inclusion method^1^ and protease supplementation on relative weights of the crop, proventriculus (Prov.), gizzard (Giz.), duodenum (Duo.), jejunum (Jej.) and ileum (Ile.), and the gizzard pH and jejunal digesta viscosity (cP) of broilers.

Barley inclusion method	Protease	Relative weights^2^, g/kg body weight						Gizzard pH^3^	Jejunal digesta viscosity^2^
		Crop	Prov.	Giz.	Duo.	Jej.	Ile.		
Fine	–	2.73	4.10	7.89	3.37	7.02	6.62	3.70	3.77
	+	2.75	3.72	8.23	3.48	7.20	7.00	3.72	3.51
									
Coarse	–	2.57	4.14	8.39	3.10	6.51	6.14	3.48	3.55
	+	2.49	3.87	7.88	3.32	6.65	6.45	3.59	3.40
									
Whole barley	–	2.31	3.03	9.76	3.13	6.47	6.05	3.02	3.53
	+	2.42	3.27	10.60	3.13	6.39	6.02	3.03	3.58
SEM		0.146	0.270	0.336	0.164	0.275	0.264	0.151	0.188
Main effects									
Barley inclusion method									
Fine		2.74^a^	4.01^a^	8.13^b^	3.43	7.11^a^	6.81^a^	3.71^a^	3.64
Coarse		2.53^ab^	3.91^a^	8.06^b^	3.21	6.58^ab^	6.29^ab^	3.54^a^	3.47
Whole barley		2.36^b^	3.15^b^	10.18^a^	3.13	6.43^b^	6.04^b^	3.02^b^	3.56
									
Protease	–	2.54	3.76	8.68	3.20	6.67	6.27	3.40	3.62
	+	2.55	3.62	8.90	3.31	6.75	6.49	3.45	3.50
*P-*value									
Barley inclusion method		0.049	0.006	0.001	0.195	0.045	0.020	0.001	0.680
Protease		0.895	0.546	0.424	0.403	0.723	0.313	0.694	0.460
Barley inclusion method × Protease		0.818	0.477	0.144	0.799	0.877	0.710	0.930	0.708

^a, b^ Means in a column not sharing a common letter are significantly different (*P* < 0.05).

1 Fine and coarse grades were achieved using screen sizes of 2.5 and 8.0 mm, respectively.

2 Each value represents the mean of 6 replicates (2 birds per replicate).

3 Each value represents the mean of 6 replicates (2 gizzards per replicate, 3 pH readings per gizzard).

## 4 Discussion

Determination of enzyme activity ensures that the added enzyme product is present and active in the feed, particularly where stability is an issue. In the current study, the average enzyme recovery for endo-1,4-β-xylanase and phytase was 155% and 201%, respectively. The endogenous enzyme activity within the grain and contaminant side activities that are neither listed nor assayed have presumably played significant roles in the recovery responses observed. Therefore, a method that only detects the exogenous product and not that of cereal origin is recommended to avoid any over-estimation in enzyme activity analysis (O'Neill et al., 2014; Bedford, 2018).

The current results showed that the coarse grinding of barley increased the relative proportion of particles >1,000 μm compared to fine grinding (77.5% vs. 28.7%). Pelleting reduced the relative proportion of large particles (>1,000 μm) in FB, CB and WB diets by 65.8% (from 41.5% to 14.2%), 59.2% (from 47.6% to 19.4%) and 52.3% (from 49.7% to 23.7%), respectively. Pelleting-induced particle size reduction was more pronounced in particles >2,000 μm, with corresponding reductions of 92.9%, 90.2% and 75.7% in FB, CB and WB diets, respectively. Due to the narrow gap between the pellet rolls and the pellet die and the frictional force inside the die holes, the pelleting process might have cracked the whole barley grains and further reduced the size of the larger particles, consequently minimising the differences in the particle size distribution between FB, CB and WB diets (Péron et al., 2005; Amerah et al., 2007b; Abdollahi et al., 2013a; Naderinejad et al., 2016).

The FB diet in the current study had greater PDI (79.5%) than CB (76.7%) and WB diets (75.7%). Perera et al. (2020) also reported superior durability in pellets made of fine barley (82.5%) compared to those made of coarse barley (79.0%). Surface area per unit volume of grain particles is increased with the extent of grinding. It can be postulated that finer grain particles can be more susceptible to gelatinisation during pelleting process than coarse particles (Svihus et al., 2004a) and, thus, resultant pellets were more durable. Considering the effect of WB inclusion on pellet durability, poor PDI reported in WB diets contrasts with Singh et al. (2014a) who reported superior PDI in whole maize diets compared to ground maize (4.0 mm) diets, even at whole maize inclusion as high as 600 g/kg. Buchanan and Moritz (2009) evaluated the inclusion of oat hulls in broiler diets and reported that pellets tended to break at oat hull contact points. Whilst NSH barley hulls are finely ground in the FB diets, CB and WB diets may have larger hulls causing more pellet breakages at hull contact points leading to poorer PDI.

In the present study, compared to birds fed FB diets, WG of birds fed CB and WB diets improved by 31 and 42 g/bird, respectively. Even though only a tendency (*P* = 0.078) was reported, birds fed CB and WB diets consumed more feed by 31 and 27 g/bird, respectively, compared to those offered FB diets. This observation suggests that increased WG may be partly attributable to higher FI in birds fed CB and WB diets. Abdollahi et al. (2016) compared 250 g/kg ground vs. whole wheat in pelleted broiler diets and reported 27 g/bird higher WG in whole wheat fed birds, without any effect on FI.

Enhanced energy utilisation and consequently better feed efficiency because of whole grain feeding to broilers, in addition to generating more developed and functional digestive tract and gizzard, have been previously reported (Wu et al., 2004; Abdollahi et al., 2016). Incorporation of WB reduced F/G by 2.7 points compared to the FB diets in the current study, a finding that might be explained by the presence of larger particles and higher GMD in WB diets than the FB diets (190 μm). In partial agreement to the current findings, Wu et al. (2004) reported no difference in FI and WG of birds fed ground wheat vs. pre-pelleting whole wheat incorporated diets, but an improved F/G of 6.5 points in birds fed whole wheat diets. Perera et al. (2020), comparing fine and coarse (2.0 and 8.0 mm, respectively) particle sizes of NSH barley (550 g/kg), reported that the effects of particle size existed even after pelleting and improved F/G of birds fed coarse barley diets by 2.1 points. Therefore, it is reasonable to speculate that when the particle size differences are preserved after pelleting, diets containing coarser particles are most likely to improve feed efficiency of broilers (Lentle et al., 2006; Perera et al., 2020). Moreover, the increases in the relative gizzard weight in the current study paralleled the improvements in F/G, confirming the beneficial influence of a functional gizzard in enhancing feed efficiency.

Facilitated by the functionality of well-developed gizzard (Svihus et al., 1997; Hetland et al., 2002) and lower gizzard pH (Svihus, 2011a), birds fed WB diets had higher digestibility of DM, N, Ca and GE by 7.65%, 8.61%, 30.0% and 7.15%, respectively, compared to birds fed FB diets. In contrast, Moss et al. (2017) compared the inclusion of ground vs. whole barley (125 g/kg) and reported lower ileal digestibility of N in birds fed whole barley (0.739 vs. 0.717), despite the well-developed gizzard. Abdollahi et al. (2016) reported no influence of replacing ground wheat with whole wheat on the ileal digestibility of N, starch and fat in broiler starters. Nevertheless, despite having gizzard weights and pH similar to birds fed FB diets, birds offered CB diets were reported with 7.14%, 5.14%, 29.8% and 6.83% higher CAID of DM, N, Ca and GE, respectively. Larger particles reduce the digesta passage rate through the gizzard (Nir et al., 1994), and are retained longer than finer particles in the digestive tract (Amerah et al., 2007a). The enhanced nutrient utilisation in birds fed CB diets, therefore, was due likely to the increased exposure time of nutrients to digestive enzymes (Amerah et al., 2011a). Comparing fine and coarse barley in pelleted barley-based diets, Perera et al. (2020) reported 3.1%, 3.2%, and 4.3% greater ileal digestibility of DM, N, and fat, respectively, in birds fed coarse barley diets, that was mainly attributed to the functional gizzards and lower gizzard pH in response to coarse barley.

Gizzard has been recognised as a key site for regulating the starch digestibility by preventing starch overload into the lower gut and a positive correlation between gizzard weight and starch digestibility has been reported (Svihus, 2011b). Moss et al. (2017) also reported an increase in ileal starch digestibility parallel to the increase in the relative gizzard weight in broilers fed 125 g/kg whole barley. In the current study, however, despite the larger gizzards and lower gizzard pH in birds fed WB diets, no influence of BIM on starch utilisation was observed. Perera et al. (2020), who compared the different particle sizes of the same NSH barley, also reported a lack of barley particle size effect on starch digestibility, despite heavier gizzards in birds fed coarse barley. Similarly, Svihus et al. (2004b) reported that 500 g/kg pre-pelleting replacement of ground wheat with whole wheat failed to show any improvement in starch digestibility. It has been suggested that the response of starch digestibility to coarseness of the diet or whole grain feeding is likely related to a complex array of confounding factors such as grain type (Carré, 2004), grain hardness (Carré et al., 2002), and feed form (Naderinejad et al., 2016).

In the present study, fat digestibility of the birds fed CB diets was superior to FB and WB fed birds. In agreement, Perera et al. (2020) reported 4.3% increase in fat digestibility attributed to the functional gizzards in birds fed coarse barley compared to those fed fine barley diets. However, given that birds fed WB diets in the current study had well-developed gizzards, the lack of WB incorporation on fat digestibility is difficult to explain.

The birds fed CB and WB diets showed an average of 30% greater Ca digestibility than those fed FB diets. Improved utilisation of Ca due to coarse grinding has been previously reported in studies comparing different maize particle sizes (Kilburn and Edwards, 2001; Amerah and Ravindran, 2009; Naderinejad et al., 2016). Comparing fine and coarse barley particle sizes, Perera et al. (2020) reported a numerically higher Ca digestibility in birds fed coarse barley particles (0.385 vs. 0.347). Most phytate–mineral complexes are soluble at pH lower than 3.5 and become insoluble at pH values between 4 and 7 (Champagne, 1988; Selle et al., 2000). The lower gizzard pH values in birds fed CB and WB diets (3.54 and 3.02, respectively), might partly explain the higher Ca digestibility than the FB diets in the current study. Moreover, it has been hypothesised that the larger particle size led to longer transit time, enhancing mineral digestion and absorption (Amerah et al., 2007a). In the present study, birds fed WB diets had a lower CAID of P compared to those fed FB and CB diets; a finding that is not readily explainable, and in contrast to studies by Kasim and Edwards (2000) and Kilburn and Edwards (2001) who reported an improved P digestibility in response to increasing particle size of maize from fine to coarse.

In the present study, paralleling the increases in DM and N digestibility, IDE values of CB and WB diets increased by 6.79% and 7.22%, respectively, compared to FB diets. In contrast, Amerah et al. (2011b) reported that pre-pelleting inclusion of whole wheat (100 and 200 g/kg) did not affect the apparent IDE of the diets in broiler starters. Neither the increase in AMEn from 12.68 to 12.78 MJ/kg DM in response to CB feeding reported by Perera et al. (2020), nor the increase in AMEn from 10.82 to 11.52 MJ/kg DM in response to WB inclusion (125 g/kg) in sorghum-based diets reported by Moss et al. (2017), was observed in the current study. Despite the BIM influenced the energy utilisation at the ileal level, the AMEn of diets remained unaffected by the BIM. It can be speculated that undigested nutrients available in the birds fed FB diets may have been subjected to a greater extent of microbial fermentation at the caecal level and consequently compromising any potential influence of BIM on AMEn measured at excreta level.

In the current study, compared to both FB and CB diets, feeding WB diets resulted in 20.5% reduction (3.96 vs. 3.15 g/kg BW) and 25.9% increase (10.2 vs. 8.10 g/kg BW) in relative weights of the proventriculus and gizzard, respectively. These findings agree with Taylor and Jones (2004) who reported 15.9% decrease and 14.0% increase in relative weights of proventriculus and gizzard, respectively, in response to the inclusion of whole barley replacing ground barley (200 g/kg). Moss et al. (2017) also reported that regardless of the basal diet type, whole barley (125 g/kg) generated heavier relative gizzard weights compared to ground barley in 28-d old broilers while eliminating the incidence of dilated proventriculi. Moreover, birds fed WB diets showed a lower gizzard pH that negatively correlated (*r* = −0.546, *P* < 0.001) with the relative weight of gizzard. Functional gizzards in broilers have been suggested to facilitate longer digesta retention time and continuous refluxes (Ferket, 2000; Singh et al., 2014b) that encourage the secretion of HCl, resulting in a lower pH. The inferences from the current and previous studies is that incorporation of whole barley in broiler diets has the potential to develop a more robust gizzard associated with enhanced gut integrity. The increase in relative gizzard weight in birds fed CB compared to those fed FB reported by Perera et al. (2020), however, was not observed in the current study, due probably to the differences in barley inclusion level (550 vs. 283 g/kg).

Supplementation of NSP-degrading enzymes has been reported to reduce the intestinal digesta viscosity of broilers fed barley-based diets (Bedford, 2018; Perera et al., 2019b). In the current study, BIM did not influence the intestinal digesta viscosity presumably due to the supplemental carbohydrase in the basal diet. Nevertheless, even without carbohydrase supplementation, Perera et al. (2020) reported no effect of barley particle size on jejunal digesta viscosity. Svihus et al. (1997) also reported that intestinal digesta viscosity of broilers (d 28) remained unchanged in response to replacing ground barley with whole barley.

In the present study, the relative weight of jejunum and ileum was lower in birds fed WB diets compared to those fed FB diets, which may presumably be a result of an adaptive response to the increased nutrient digestibility in birds fed WB diets (Brenes et al., 1993). Previous studies with wheat, however, have failed to show any impact of substitution of ground wheat with whole wheat on the relative weights of small intestinal segments (Jones and Taylor, 2001; Engberg et al., 2004; Wu et al., 2004).

Although the main intention of an exogenous protease addition to poultry diets is to enhance protein digestibility, the benefits of an exogenous protease extend well beyond by improving the growth performance (Kalmendal and Tauson, 2012) and utilisation of other nutrients (Selle et al., 2013; Cowieson and Roos, 2016; Cowieson et al., 2017). Kalmendal and Tauson (2012) reported that starch, fat and energy utilisation of broilers (d 34) fed wheat-based diet was improved by protease supplementation. Protein utilisation, however, showed only numerical improvements in response to protease. Cowieson et al. (2017) reported that protease addition to a maize-based diet increased not only N digestibility but also digestible energy content of the diets. Selle et al. (2013) also reported that supplemental protease in sorghum-based diets improved ileal digestibility of both N and starch. However, protease failed to have any positive effect on nutrient utilisation and growth performance in the current study, due probably to the lack of proteinous anti-nutrient such as lectins, trypsin inhibitors, antigenic proteins in the basal diet. Non-proteinous anti-nutrients present in wheat and barley may have been completely degraded by carbohydrases and phytase in the diets, leading to the disintegration of protein-matrix and consequently leaving little room for improvement by added protease.

Moreover, the dependency of enzyme responses on the quality of the diet to which they are added may also explain the lack of positive effect of protease on both nutrient digestibility and performance in the current study. The lower the ingredient quality or dietary nutrient density, the greater the magnitude of improvements with added enzymes (Ravindran, 2013). The responses of protease in improving the growth performance of broilers (d 35) when added to protein and AA-deficient diets reported by Cho et al. (2020) support this postulation. The basal diet in the current study was formulated to meet the Ross 308 strain requirements for digestible AA, reducing the capacity of exogenous protease to elicit an effect. Cowieson and Roos (2014), suggested that the inherent digestibility of AA in the control diet with no protease supplementation is the primary predictor of protease effect. These researchers, conducting a meta-analysis study of the effect of same protease as the current study, predicted an average of 5.5% increase in AA digestibility by protease supplementation when the control diet AA digestibility is 70%. However, the magnitude of digestibility response to protease reduced rapidly as control AA digestibility increased from 70% to 80%. The N digestibility of the basal diet without protease supplementation in the current study was 74.8%, which can further explain the absence of protease effect.

## 5 Conclusions

The findings of the present study showed that CB and WB (NSH barley at 283 g/kg) inclusion in wheat-based broiler starter diets is beneficial to bird performance, as indicated by higher weight gain and feed efficiency, particularly in broilers fed diets containing WB. The increases in digestibility of nutrients may have been responsible for the improved growth performance of broilers fed CB and WB diets. The lack of protease effect on nutrient utilisation and performance of the birds, may hypothetically mirror a low potential for further improvements, due possibly to the fact that the basal diet was formulated to meet the digestible AA requirements of birds, high inherent digestibility of protein in the control diet, and/or the presence of exogenous carbohydrase and phytase.

## Author contributions

**L. M. Tari**: Animal trial, Data collection and evaluation, Laboratory and Statistical analysis, Writing; **N. Perera**: Animal trial, Data collection and evaluation, Manuscript review; **F. Zaefarian**: Methodology, Data evaluation, Manuscript review; **M. R. Abdollahi**: Study design, Feed formulation, Data evaluation, Critical manuscript review; **A. J. Cowieson**: Data evaluation, Manuscript review; **V. Ravindran**: Data evaluation, Manuscript review.

## Declaration of competing interest

We declare that we have no financial and personal relationships with other people or organizations that can inappropriately influence our work, and there is no professional or other personal interest of any nature or kind in any product, service and/or company that could be construed as influencing the content of this paper.
